# The association between microvascular obstruction by CMR and the index of microcirculatory resistance assessed invasively

**DOI:** 10.1186/1532-429X-16-S1-P212

**Published:** 2014-01-16

**Authors:** Elisa McAlindon, Tom Johnson, Julian Strange, John Edmond, Jessica Harris, Maria Pufulete, Andreas Baumbach, Chiara Bucciarelli-Ducci

**Affiliations:** 1 CMR Unit, NIHR Bristol Cardiovascular Biomedical Research Unit, Bristol Heart Institute, Bristol, UK; 2 Cardiology, Bristol Heart Institute, Bristol, UK; 3 Clinical Trials and Evaluation Unit, University of Bristol, Bristol, UK

## Background

Microvascular obstruction (MVO) by CMR is a predictor of poor prognosis following acute myocardial infarction. The index of microcirculatory resistance (IMR) is an invasive measurement of the microcirculation that has recently been shown to predict poor long-term outcomes [1]. The aim of the study was to determine the association between MVO by CMR and the IMR measured at the time of primary percutaneous intervention (PPCI) in patients with ST-elevation myocardial infarction (STEMI).

## Methods

48 patients were prospectively recruited to the study. Inclusion criteria were: presentation with STEMI within 12 hours of symptoms and TIMI flow I or 0 in the infarct related artery. Patients needed to proceed with PPCI in a large epicardial artery. Patients with contraindications for CMR were excluded from the study. IMR was performed using a pressure thermistor wire (Certus, St Jude) at maximal hyperaemia using adenosine, and following stent insertion. CMR was performed at day 2 following STEMI. We used an IMR cut off of 40 that has been shown to predict prognosis(1), and IMR quartiles to investigate an association between MVO and IMR. The difference between IMR quartiles was assessed using the Kruskal-Wallis test. The difference between MVO in patents with an IMR more or less than 40 was assessed using the Mann-Witney test. All patients provided informed written consent and the study was approved by the regional ethics committee.

## Results

**Figure 1:**
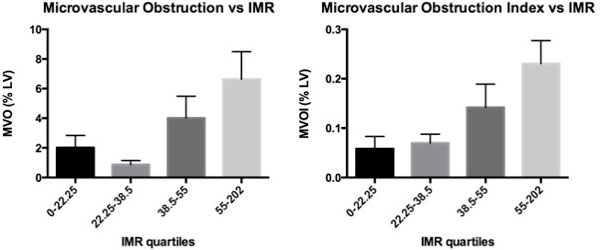
**a) MVO vs. IMR (p = 0.007) Figure 1b) MVOI vs. IMR (p = 0.003)**.

**Figure 2:**
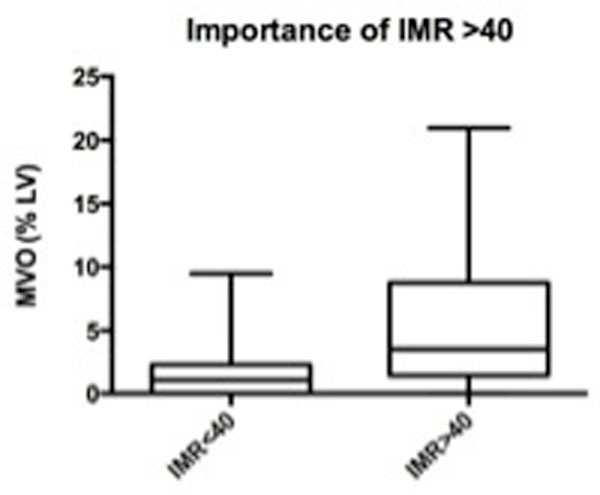
**MVO predicted by IMR > and < 40 (p = 0.0003)**.

The median IMR was 38.5 (range 9 to 202). The median MVO was 1.9% LV (range 0 to 21.0% LV). There was a significant increase in MVO as IMR increased (p = 0.007) (Figure 1a). This is more pronounced when MVO was indexed to infarct size (MVO/Infarct size, MVOI) (p = 0.003) (Figure 1b). The IMR cut-off of 40 significantly predicted the presence of MVO on CMR (p = 0.0003) (Figure 2).

## Conclusions

IMR is significantly associated with MVO on CMR day 2 following STEMI. This study provides an invasive functional insight into MVO measured non-invasively by CMR.

## Funding

This study was funded by the David Telling Charitable Trust and the NIHR Bristol Cardiovascular Biomedical Research Unit.
